# Protocol to separate small and large extracellular vesicles from mouse and human cardiac tissues

**DOI:** 10.1016/j.xpro.2024.102914

**Published:** 2024-02-21

**Authors:** Wenjing Liang, Rita H. Najor, Åsa B. Gustafsson

**Affiliations:** 1 Skaggs School of Pharmacy and Pharmaceutical Sciences, University of California San Diego, La Jolla, CA 92093, USA

**Keywords:** Health Sciences, Microscopy, Signal Transduction

## Abstract

Extracellular vesicles (EVs) are secreted by cells under various conditions and can contribute to the disease progression in tissues. Here, we present a protocol to separate small and large EVs from mouse hearts and cardiac tissues collected from patients. We describe steps for utilizing enzymatic digestion for release of EVs from interstitial space followed by differential centrifugation and immunoaffinity purification. The isolated EVs can be used for various experiments to gain insight into their *in vivo* functions.

For complete details on the use and execution of this protocol, please refer to Liang et al. (2023).^1^

## Highlights

• Detailed protocol for isolating EVs from human and mouse cardiac tissues
• Describes processing and digestion of human and mouse tissue to release EVs
• Step-by-step details on separating and purifying large and small EVs
• Isolated EVs are suitable for characterization and functional experiments

## Abstract (editor highlights)

Publisher’s note: Undertaking any experimental protocol requires adherence to local institutional guidelines for laboratory safety and ethics.

## Graphical abstract

**Figure:**
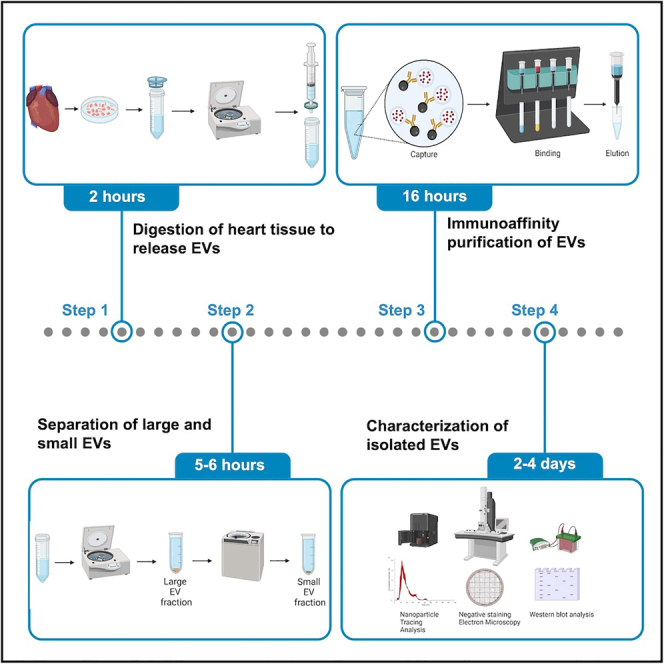

## Before you begin

To isolate EVs from mouse hearts and human cardiac tissue, we applied two sequential methods: differential centrifugation to separate the large (∼200–800 nm) and small (∼50–200 nm) EV fractions followed by immunoaffinity purification using antibodies conjugated to magnetic beads for further separation of the vesicles.

1. Before you begin the isolation procedure, it is important that all the essential buffers have been prepared and chilled to 4°C.
2. You also need to have sterilized tools, tissue culture plates, and various tubes set up at the appropriate workstations. Keep everything on ice or at 4°C, unless stated otherwise.
3. Centrifuges and rotors should be prechilled to 4°C for 12–18 h before they are used.
4. Prepare Digestion Buffer (Hanks Balanced Salt Solution (HBSS) plus Liberase DH (200 μg/mL) and DNase I (20 U/mL)) on the day of the procedure and maintain on ice until use.

***Note:*** All incubations are performed at 4°C unless otherwise noted.

### Institutional permissions

All animal experiments must be performed following the Guidelines of National Institutes of Health on the Use of Laboratory Animals and with the approval of the Institutional Animal Care and Use Committee at your research institution. Human heart biopsies require an Institutional Review Board approval.

## Key resources table

**Table:** 

REAGENT or RESOURCE	SOURCE	IDENTIFIER
**Antibodies**		

CD81 (1:1,000), for mouse tissue	Cell Signaling Technology	10037
CD81 (1:1,000), for human tissue	Cell Signaling Technology	56039
CD63 (1:500), for mouse and human tissues	Thermo Fisher Scientific	PA5-92370
CD9 (1:1,000), for mouse and human tissues	System Biosciences	EXOAB-CD9A-1
Tsg101 (1:1,000), for mouse and human tissues	Abcam	Ab83
Alix (1:1,000), for mouse and human tissues	Cell Signaling Technology	2171
Goat anti-rabbit IgG (H + L) HRP secondary antibody (1:5,000)	Thermo Fisher Scientific	G21234
Goat anti-mouse IgG (H + L) HRP secondary antibody (1:5,000)	Thermo Fisher Scientific	G21040

**Chemicals, peptides, and recombinant proteins**		

Pierce Bradford Plus Protein Assay Kit	Thermo Fisher Scientific	23236
Ponceau S staining solution	MilliporeSigma	P7170-1L
Hank’s balanced salt solution (HBSS)	MilliporeSigma	H6648-500ML
Dulbecco’s phosphate-buffered saline (PBS)	Thermo Fisher Scientific	14200075
Roche Liberase DH	MilliporeSigma	5401089001
Roche DNase I	MilliporeSigma	04716728001
EV Isolation Kit pan, human	Miltenyi Biotec	130-110-912
EV Isolation Kit pan, mouse	Miltenyi Biotec	130-117-039
ECL western blotting detection reagent	Thermo Fisher Scientific	34076
NuPAGE MOPS SDS running buffer (20×)	Thermo Fisher Scientific	NP0001
4× LDS sample buffer	Thermo Fisher Scientific	NP0008
10× Tris/glycine buffer	Bio-Rad	1610771
Tris	Bio-Rad	161-0719
NaCl	Thermo Fisher Scientific	S671-3
EGTA	MilliporeSigma	E4378
EDTA	MilliporeSigma	E4884
Triton X-100	MilliporeSigma	T9284
Roche cOmplete protease inhibitor	MilliporeSigma	11873580001
Roche PhosSTOP	MilliporeSigma	4906837001
Precision Plus protein standards	Bio-Rad	161-0373
Tween 20	Thermo Fisher Scientific	BP337-500
Methanol	Thermo Fisher Scientific	A452-4
DTT	Bio-Rad	1610611
Uranyl acetate dihydrate	Ladd Research Industries	23620
Fatal-Plus	Vortech Pharmaceuticals	NDC 0298-9373-68

**Experimental models: Organisms/strains**		

Mouse: C57BL/6J	The Jackson Laboratory	Strain #000664

**Software and algorithms**		

ImageJ/Fiji software	Schneider et al.^2^	Fiji Downloads (imagej.net)

**Others**		

Orbital shaker	VWR	VWR Incubating Shaker
Benchtop centrifuge	Eppendorf	Eppendorf Centrifuge 5810R
Ultracentrifuge	Beckman Coulter	Optima L-90K Ultracentrifuge
Type 32 Ti swinging bucket rotor	Beckman Coulter	369650
Ultra-clear centrifuge tubes	Beckman Coulter	344058
MACS separator	Miltenyi Biotec	130-042-602
MACS MultiStand	Miltenyi Biotec	130-042-303
Transmission electron microscope	JEOL	JEM-1400Plus
NanoSight NS300	Malvern Panalytical	NS300
ChemiDoc imaging system	Bio-Rad	ChemiDoc XRS+
Corning TC-treated culture dishes, 100 mm	Thermo Fisher Scientific	430293
Bioland Scientific LCC 35 mm cell culture dish	Thermo Fisher Scientific	706001
Corning Costar 6-well clear TC-treated multiple well plates	Thermo Fisher Scientific	3506
50 mL centrifuge tubes	BioPioneer	CNT-50
Olympus 1.7 mL microtubes	Genesee Scientific	22-281
Millex-MP filter unit (0.22 μm syringe filter)	MilliporeSigma	SLMP025SS
0.8 μm filter unit	GE Healthcare	10462240
70 μm cell strainer	Falcon	352350
NuPAGE 12%, Bis-Tris, 1.0 mm, mini protein gels	Thermo Fisher Scientific	NP0341box
Nitrocellulose membrane	Genesee Scientific	84-875
Whatman filter paper	VWR	3030-6461

## Materials and equipment

This protocol uses a Beckman Coulter Optima L-90K Ultracentrifuge with a Type 32 Ti Swinging bucket rotor to pellet EVs. A JEOL JEM-1400Plus transmission electron microscope is used for imaging of negatively stained EVs, and a NanoSight NS300 is used for the nanoparticle tracking analysis. Similar equipment are available from other manufacturers and will also work for this protocol.

**Table:** Digestion Buffer (2 mL)

Reagent	Final concentration	Amount
HBSS	1×	1,916 μL
Liberase DH	200 μg/mL	80 μL
DNase I	20 U/mL	4 μL

Must be prepared fresh.

***Alternatives:*** We use commercially prepared HBSS from Sigma. It is also available from other companies such as Thermo Fisher Scientific (Cat.# 14025092). HBSS can also be prepared in the lab.

***Alternatives:*** We always use Liberase DH to digest the heart tissue. This is an enzyme blend consisting of highly purified Collagenase I and Collagenase II. It is possible to purchase these enzymes from other sources for the tissue digestion. Also, Collagenase II alone is often used to digest hearts for the isolation of adult cardiac myocytes^3^ and could potentially be used to release EVs.

**Table:** Western Blot Lysis Buffer

Reagent	Final concentration	Amount
1 M Tris-HCl (pH 7.4)	50 mM	2.5 mL
5 M NaCl	150 mM	1.5 mL
0.1 M EGTA	1 mM	0.5 mL
0.1 M EDTA	1 mM	0.5 mL
20% Triton X-100	1%	2.5 mL
Complete Protease Inhibitor	1×	1 tablet
PhosSTOP	1×	1 tablet
ddH_2_O	N/A	42.5 mL
**Total**	**N/A**	**50 mL**

Can be stored at 4°C for 1 week without Protease inhibitors and PhosSTOP.

**Table:** Transfer Buffer

Reagent	Final concentration	Amount
10× Tris/glycine buffer	1×	100 mL
Methanol	20%	200 mL
ddH_2_O	N/A	700 mL
**Total**	**N/A**	**1,000 mL**

Can be stored at 4°C for 1 month.

**Table:** TBS-Tween 20 (TBST) (100 mL)

Reagent	Final concentration	Amount
1 M Tris-HCl (pH 7.4)	50 mM	5 mL
5 M NaCl	150 mM	3 mL
5% Tween 20	0.05%	1 mL
ddH_2_O	N/A	91 mL
**Total**	**N/A**	**100 mL**

Can be stored at 4°C for 1 month

## Step-by-step method details

### Collection and digestion of cardiac tissue (step 1)

#### Harvest hearts from mice

**Timing: 10–20 min per mouse**

This section describes how to harvest hearts from mice for separation of EVs.

1. Allocate a clean and uncluttered work area for the surgery.
  a. Clean the surgical area with 70% ethanol and apply a clean absorbent pad over the surface where the surgery will be performed.
  b. Place the surgical tools on the pad.
2. Euthanize the mouse with Fatal-Plus (Pentobarbital Sodium, 100 mg/kg) or by other institutionally accepted and approved method of euthanasia.
3. Place the mouse in a supine position and spray the chest with 70% ethanol.
  a. Quickly open the thoracic cavity to collect the heart. For a detailed description of the surgery see Pinto et al. (2013).^4^
  b. Use sharp scissors to nick the right atrium, allowing blood to drain from the heart, followed by promptly excising the heart from the chest.
  c. Immerse the heart in ice-cold HBSS in a 100 mm dish on ice. Gently squeeze the heart with forceps to push out any remaining blood in the chambers.
  d. Keep the dish on ice and trim off any remaining atrial tissue using the surgical scissors.

***Note:*** Only ventricles are used for the separation of EVs since they are the main muscles responsible for pumping blood out of the heart and become dysfunctional in cardiovascular disease.

**Pause point:** Hearts can be snap frozen in liquid nitrogen and stored at −80°C for up to 6 months.

#### Preparation of human heart tissue

**Timing: 5–10 min per heart sample**

This section describes how to prepare cardiac biopsies that have previously been collected from patients for separation of EVs.

4. Heart tissues are collected from patients undergoing heart transplants and frozen for later use.
5. Keep the frozen cardiac tissues on dry ice as you move them from −80°C to the bench.
6. Trim the frozen cardiac tissues if needed with a sharp razor blade.

**CRITICAL:** A minimum of 100–120 mg of tissue is needed to obtain sufficient number of EVs for subsequent analysis.

7. Add each tissue to a 100 mm cell culture dish on ice containing ice-cold HBSS.
8. Maintain on ice for dissection.

***Optional:*** Save 50–100 μg of the heart tissues for Western Blot analysis. We always save a small piece of heart tissue so that we can include the whole heart lysate in any Western Blot analysis performed on the isolated EVs. We usually load 20–30 μg of heart lysate per well for the Western Blot experiments. This allows us to confirm expression levels of EV markers in the heart tissue being analyzed. It also serves as a positive control for the antibodies.

#### Digestion of heart tissue (mouse or human)

**Timing: 60 min**

This section describes how to perform the enzymatic digestion of heart tissue to release the EVs from the interstitial space.

9. Add 2 mL of Digestion Buffer (HBSS supplemented with Liberase DH (200 μg/mL) and DNase I (20 U/mL)) to a 6-well or 35 mm plate on ice.
10. Transfer 100–120 mg fresh or frozen cardiac tissue into the plate containing Digestion Buffer.

***Note:*** It is important to weigh the heart tissue before adding it to the Digestion Buffer to ensure uniformity in between the groups being compared. We found that a minimum of 100 mg of mouse heart tissue is needed for the separation of a sufficient number of vesicles for subsequent analysis. However, the protocol can be scaled up by combining two hearts if more EVs are needed.

**Figure 1:**
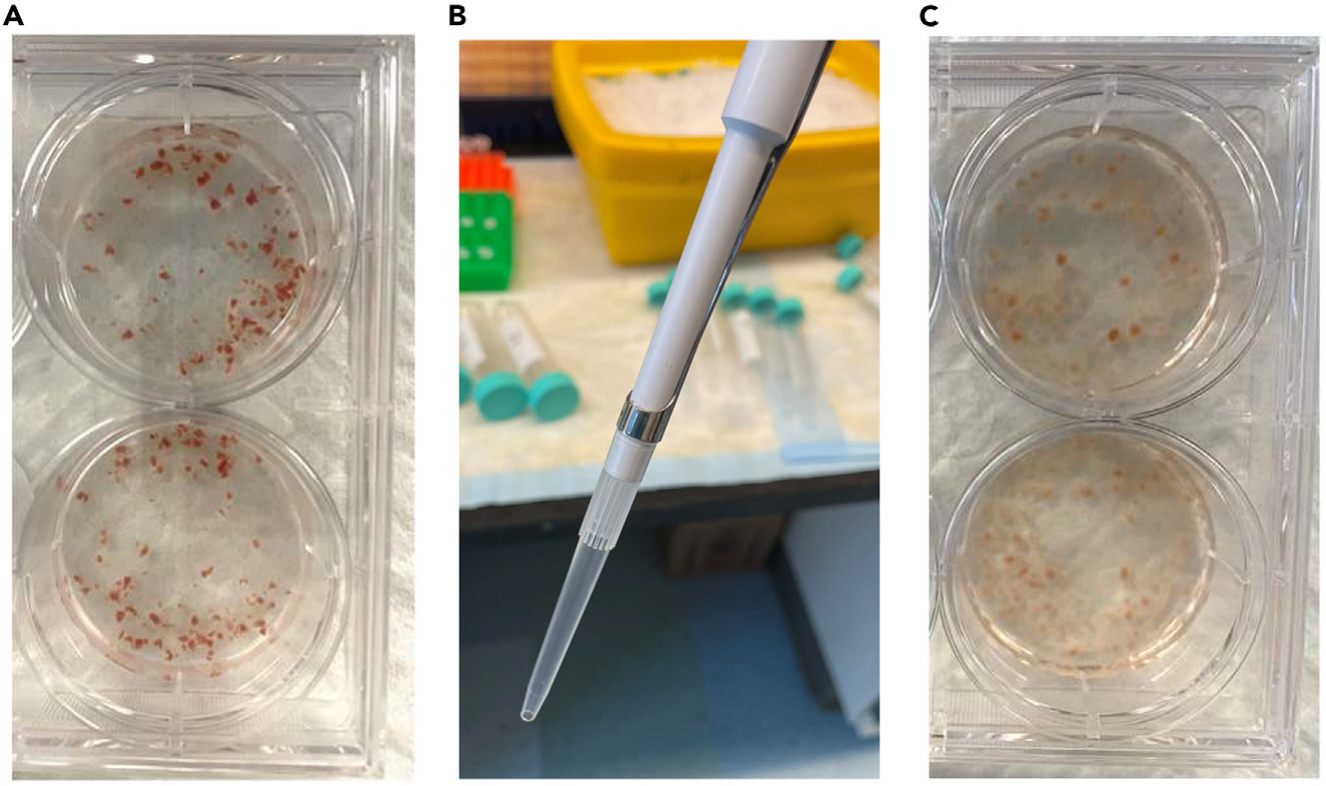
Digestion of hearts to release EVs from the interstitial space (A) Heart tissue is minced into 2 × 2 × 2 mm pieces in Digestion Buffer. (B) P1000 pipette tip is trimmed at the end before pipetting the digested tissue. (C) Heart tissue after digestion and gentle pipetting.
12. Transfer the minced tissue to a 37°C Orbital shaker incubator and incubate for 45 min under mild agitation (50 rpm).
13. Use a P1000 pipette with the tip trimmed at the end (∼2 mm) to avoid blockage.
14. Gently pipette the digested heart tissue up and down 20 times to release the EVs from the interstitial space (Figure 1B).

***Note:*** The Digestion Buffer should become cloudy after the pipetting (Figure 1C).

11. Mince the tissue into 2 × 2 × 2 mm pieces using microsurgery scissors in Digestion Buffer while on ice (Figure 1A).

**CRITICAL:** It is important to not over digest the tissue or pipette too vigorously after the digestion as this can cause rupture of fragile cells (especially in a diseased heart) and lead to release of intracellular vesicles. The level of digestion can be tested by carefully pipetting the tissue during incubation in the Digestion Buffer. If tissue is still firm and doesn’t easily break up, then additional time is needed. Although the enzymatic digestion of the connective tissue is gentle, extended time could result in digestion of surface proteins on both EVs and cells.

***Note:*** With optimization of the digestion time, this protocol can be adapted for use to separate EVs from other tissues, such as skeletal muscle, kidney, and adipose tissue, as well as animal species such as rats. Factors that will affect digestion time of tissues include age and disease state. More fibrotic tissues require longer digestion times.

### Separation of small and large EV fractions (step 2)

**Timing: 5–6 h**

**Figure 2:**
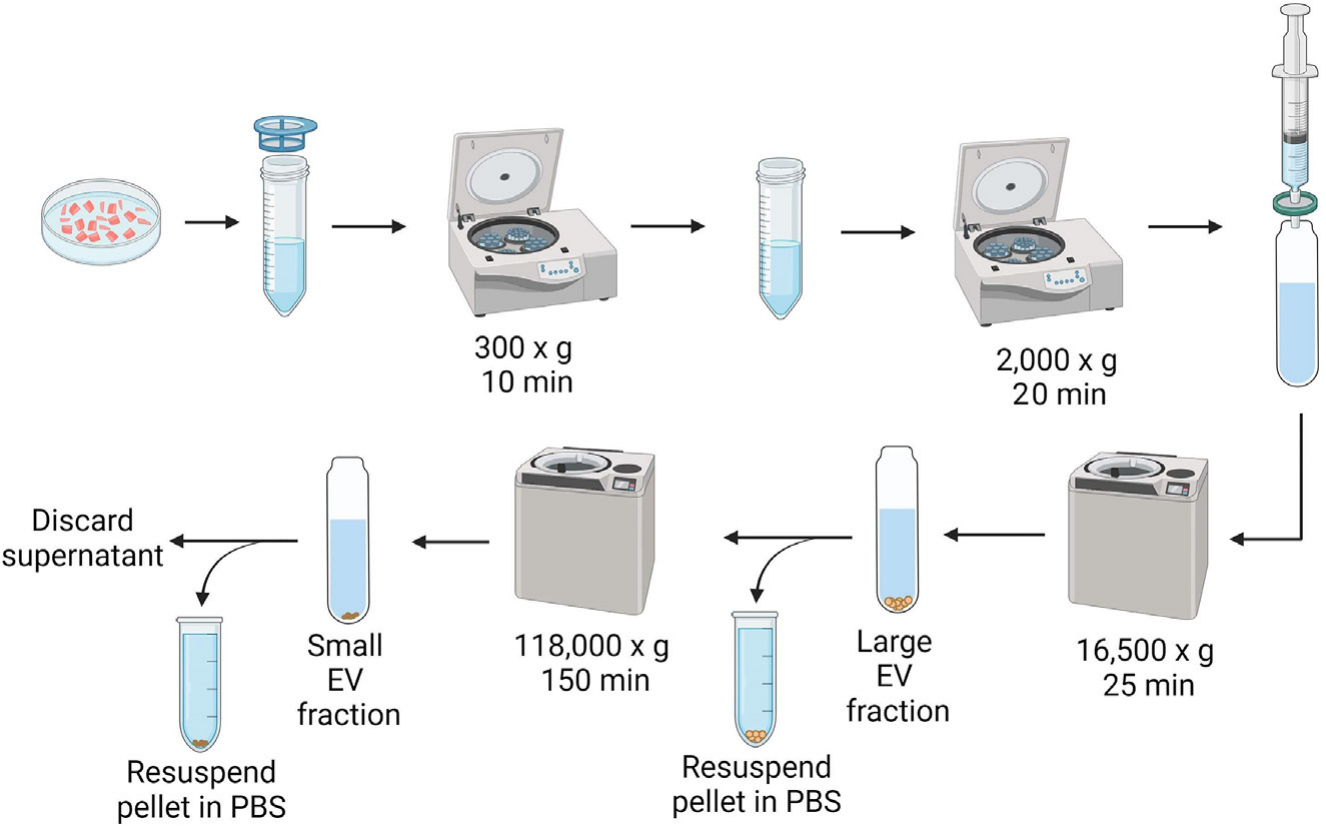
Overview of differential centrifugation steps to separate small and large EVs

This section describes how to separate the small and large EVs that have been released from interstitial space of the digested cardiac tissue using differential centrifugation (Figure 2).

15. Place a 70 μm cell strainer over a 50 mL centrifugation tube and filter the digested heart tissue through the strainer.
16. To collect any remaining digested material, rinse the plate with 5 mL of HBSS and pour it over the strainer. Once the digested material has passed through the filter via gravity, the supernatant is ready for centrifugation.
17. Centrifuge the filtered material at 300 × *g*, 4°C for 10 min in the Eppendorf Centrifuge 5810R to pellet cells and larger debris.
18. Transfer the supernatant to a clean 50 mL centrifugation tube and be careful not to disturb the pellet.

***Note:*** Instead of pipetting, it is better to carefully pour the supernatant when transferring to the new tube to avoid disturbing the pellet. With a swinging bucket rotor, the EV pellet will be at the bottom of tube after centrifugation. Centrifugation using a fixed angle rotor will result in a pellet on the side of the tube. Please see Hu et al. 2021^5^ for technique on how to decant the supernatant if a fixed angle rotor is used.

19. Centrifuge the supernatant at 2,000 × *g*, 4°C for 20 min in the Eppendorf Centrifuge 5810R to remove smaller cells and potential apoptotic bodies.
20. Set up a 10 mL syringe attached to a 0.8 μm cellulose acetate syringe filter system on top of an ultra-clear centrifugation tube and filter the supernatant into the tube.
21. Add an additional 3 mL of HBSS buffer to dilute the solution and avoid clogging the filter.

***Note:*** The filtering step ensures that all EVs in the supernatant are <800 nm when starting the next step in the protocol.

22. Carefully balance the tubes.
  a. Weigh each tube.
  b. Add additional cold HBSS to one of the tubes to make sure that the weights are equal.
23. Prechill the ultracentrifuge rotor and adaptors for 12–18 h at 4°C.
24. Centrifuge the filtered supernatant at 16,500 × *g*, 4°C for 25 min to collect the large EV fraction.
25. Decant the supernatant and the pellet represents the large EV fraction (∼200–800 nm).

***Note:*** It is not necessary to use the ultracentrifuge for step #24 when isolating large EV fraction. Any centrifuge that can hold larger tubes (at least 8 mL) and spin at the required g force (16,500 × *g*) is sufficient.

***Note:*** The k-factor for the 32 Ti swinging bucket rotor at max speed is 204.

26. Carefully pour the supernatant into a new ultra-clear centrifugation tube while making sure not to dislodge the large EV pellet.
27. Resuspend the large EV pellet in 500 μL ice-cold PBS, transfer to a 1.7 mL Eppendorf tube and maintain on ice while isolating the small EVs.
28. Weigh the tubes containing the supernatant with small EVs to make sure they are balanced and add additional HBSS as needed.
29. Spin the supernatant at 118,000 × *g*, 4°C for 150 min to collect the small EVs (∼50–200 nm).
30. Once the centrifugation is complete, discard the supernatant.

***Note:*** For the ultracentrifugation step, it is essential that all tubes are balanced and filled with liquid nearly to the top, leaving a slight gap of about 0.5–1 cm. Insufficient filling can lead to collapse of the tube during centrifugation.

31. Place the tubes upside down on a paper towel for approximately 1–2 min to eliminate any remaining HBSS.
  a. Use a Kim Wipe to fully soak up the remaining liquid from the inner tube surface.
  b. Resuspend the small EV pellets in 500 μL ice-cold PBS.
  c. Transfer to 1.7 mL Eppendorf tubes on ice.

### Immunoaffinity purification of EVs (step 3)

**Timing: 14–16 h**

**Figure 3:**
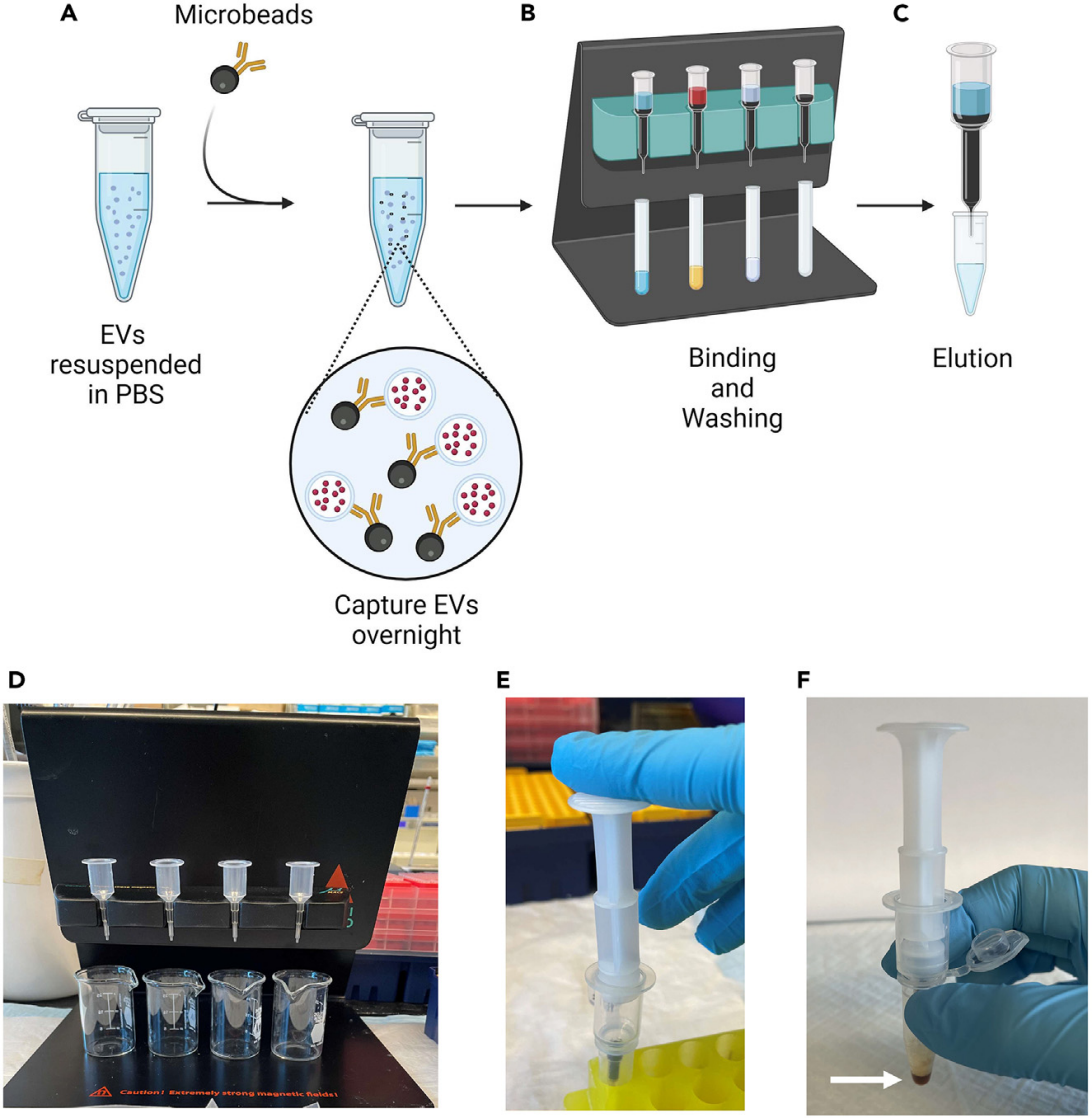
Overview of immunoaffinity purification of EVs (A) MicroBeads are added to large and small EV fractions and incubated for 12–16 h at 4°C. (B) Magnetically labeled EVs are retained within the column in the magnetic field. (C) EVs are eluted by removing columns from the magnetic field and adding PBS or Western Blot lysis buffer. A plunger is used to push through liquid and elution of EVs into an Eppendorf tube. (D) MACS columns attached to a MACS MultiStand. (E) A plunger attached to a column for elution of EVs. (F) EVs eluted in Western Blot lysis buffer.

This section describes the separation of EVs from each fraction using antibodies against specific proteins that are present on the surface of the vesicles (Figure 3). We use the EV Isolation Kit Pan (human or mouse, Miltenyi Biotec) containing magnetic MicroBeads cross linked to antibodies recognizing tetraspanin proteins CD9, CD63, and CD81 for this step. However, there are alternative antibody-conjugated beads from other companies (e.g., Thermo Fischer Scientific, Abcam, System Biosciences) that are using the same principle and can be used for the immunoaffinity purification of EVs.

32. Add 50 μL of EV Isolation MicroBeads Pan (EV Isolation Kit Pan mouse or human, Miltenyi Biotec) recognizing the specific tetraspanin proteins, CD9, CD63 or CD81, to each tube containing 500 μL of EVs from steps #27 and #31.
33. Transfer tubes to a benchtop tube rotator (Barnstead Rotator Model 415110) at 4°C and incubate 12–16 h (Figure 3A).
34. The next morning, prewarm the Equilibration Buffer and Isolation Buffer to 18°C–20°C for 30 min.

***Note:*** These buffers are components of the EV Isolation Kit Pan from Miltenyi Biotec and should be stored at 4°C.

35. Prepare the columns. Place the MACS columns in the magnetic field of the MACS Separator attached to a MACS MultiStand (Figures 3B and 3D).
36. Add 100 μL Equilibration Buffer to the columns.
37. Rinse the columns with 3 × 100 μL Isolation Buffer.

***Note:*** Make sure no buffer is remaining in the column reservoir before adding more buffer for the next rinse. However, it is important to never let the column run dry.

38. Retrieve the samples from the tube rotator at 4°C that have been incubated 12–18 h and add the solution to the columns.

***Note:*** Magnetically labeled EVs will be retained on the columns while material not bound to magnetic beads will flow through.

39. Wash the columns with 4 × 200 μL of Isolation Buffer.

***Note:*** For each wash - make sure no buffer is remaining in the column reservoir before adding more Isolation Buffer.

40. Remove the columns from the magnetic separator and place each column onto a 1.7 mL Eppendorf tube.
41. Add 65–100 μL of Elution Buffer (can be PBS or Western Blot Lysis Buffer, depending on the experimental plan for the EVs) and immediately elute the EVs by firmly pushing down the plunger (Figures 3C and 3E).

***Note:*** The eluate contains the EVs and is used for further analysis.

**Pause point:** EVs eluted in Western Blot Lysis Buffer can be stored at −80°C for up to 1 year.

**CRITICAL:** The isolated EVs eluted in PBS should not be subjected to freeze and thaw cycles. It is advisable to use them immediately or store at 4°C for no more than 24 h. Additionally, small EVs generally exhibit greater stability than large EVs, allowing them to be more durable at 4°C.

***Note:*** We have also used differential centrifugation combined with affinity purification to separate large and small EVs from plasma and from conditioned media collected from cultured cells. However, keep in mind that the amount of EVs secreted varies between cell lines and tissues. For example, EV secretion is increased in various cancer cell lines^6^ and in cells with compromised lysosomal function.^1^ The digestion time for other tissues also needs to be optimized. We find that 45 min is sufficient to digest heart tissue, while brain tissue from mice only requires 20 min of digestion.^7^

### EVs protein quantification and particle characterization (step 4)

**Timing: 2–4 days**

**Figure 4:**
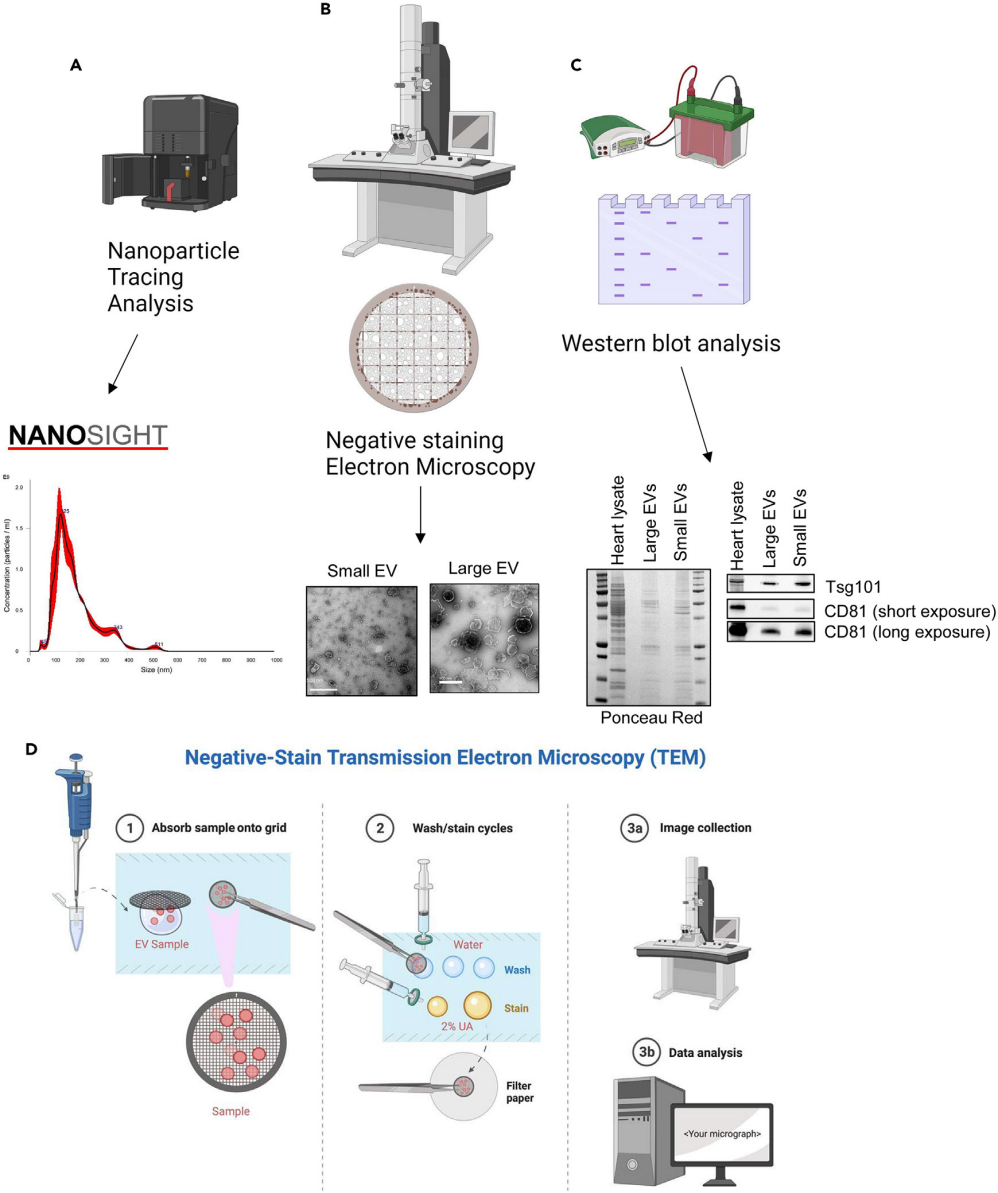
Characterization of EVs Overview of EV characterization by (A) nanoparticle tracking analysis (NTA) of small EVs, (B) negative stain electron microscopy (EM), and (C) Western Blot analysis of small and large EVs purified from a mouse heart. Ponceau Red stain shows total protein content on nitrocellulose membrane. Tsg101 and CD81 were used as EV markers. Scale bars = 500 nm. (D) Steps involved in the negative staining of EVs for EM.

This section describes three different common methods used to characterize the EVs that have been separated from the tissue (Figure 4).

#### Nanoparticle tracking analysis (NTA)

This section describes the evaluation of the isolated EVs by NTA or a similar method to confirm their concentrations and size distributions (Figure 4A). NTA can be used for particles from 10 to 1,000 nm in diameter. Detailed protocols describing the NTA analysis for EVs using the NanoSight N300 have previously been published.^8^^,^^9^

42. Elute large and small EVs from the columns (see Step #41 above) in 100 μL cold PBS.
43. Dilute 20 μL of the samples in 1 mL PBS and store on ice until ready to use.
44. Turn on the NanoSight NS300 instrument and allow it to warm up.
45. Open the NTA software on the computer that is connected to the instrument.

***Note:*** If the isolated EVs originate from larger heart samples (>100 mg), it might be necessary to dilute the samples further to ensure that the concentration aligns with the instrument's optimal measurement range to prevent excessive particle signal saturation. For a 100 mg heart sample, we dilute eluted EVs 1:50 for the NTA experiments. For larger samples, we recommend starting with a 1:100 dilution.

46. Rinse the NanoSight N300 sample chamber and tubing with 70% ethanol, followed by a rinse with PBS.
47. Add sample to a 1 mL syringe and insert into the sample inlet port of the NS300.

**CRITICAL:** Avoid introducing bubbles into the system.

48. Set up the appropriate parameters, such as camera level and detection threshold, in the NTA software to achieve the best visualization and accuracy of the EVs.

***Note:*** We always set the camera level to 13 and the detection threshold to 5.

49. Begin data acquisition, capturing three videos of particle movement, 60 s of each.
50. Once the data acquisition is complete, start the data analysis and histogram generation in the NanoSight N300 software.
51. When done, export the data (vesicle movement videos, excel spreadsheet with raw data and PDF file of report summary).
52. After each sample, flush the instrument’s sample chamber and tubing with PBS before loading the next sample.
53. When the experiment and analysis have been completed, clean the sample chamber and tubing with 70% ethanol and ddH_2_O according to the manufacturer’s guidelines (NanoSight NS300 | Characterize Nanoparticles | Product support | Malvern Panalytical).
54. When done, close the software and turn off the N300 instrument.

#### Negative staining electron microscopy (EM)

This section describes how to perform negative staining EM to confirm that the isolated EVs have retained their size and shape (Figures 4B and 4D).

55. Elute the large and small EVs from the columns (see step #41 above) in 100 μL cold PBS.
56. Perform glow discharge on the carbon-coated EM grids to make the surface less hydrophobic.
57. Apply a piece of Parafilm to the glass surface with the paper side facing up.
58. Pipet a 5–20 μL drop of the EV sample (from step #55) onto the Parafilm.
59. Place grid carbon-side down in the sample using a microsurgery tweezer, and let it sit for at least 5 min.
60. Add 3 drops of ddH_2_O using a syringe equipped with a 0.22 μm syringe filter onto the Parafilm.
61. Transfer the grid using microsurgery tweezer with the carbon side facing down to the first water drop, allowing it to rest for 30 s.
62. Move it to the second and third water drops, maintaining a 30-s pause for each.
63. Dispense 2 drops of 2% Uranyl Acetate (UA) in water onto the parafilm using a syringe equipped with a 0.22 μm syringe filter.

***Note:*** The first drop should be smaller than the second drop.

***Note:*** Uranyl acetate is a radioactive hazardous substance and should always be handled in a ventilated fume hood while wearing appropriate personal protective equipment (PPE). Dispose of waste following Institutional guidelines for hazardous materials.

64. Transfer the grid carbon-side down to the first drop of UA; hold for 3 s.
65. Follow the same procedure as above for the second larger drop of UA. Let sit for 1 min.
66. Fold a piece of filter paper in half to create a bend.
  a. Remove the grid from the second drop of UA.
  b. Pull the grid up from the solution vertically (without a slant) in order to get a large drop of UA on the grid.
67. Blot the edge of the grid to the filter paper and push down lightly for 5 s.
68. Air-dry the grid using the tweezers to hold the edge of the grid for approximately 20 min (or until no liquid is visible under light).

***Note:*** Exposing both sides of the grid to air allows for more efficient drying.

69. When the grid is dry, slide a small piece of filter paper between the tweezers to absorb any leftover UA and push out the grid.
70. Place in grid-box and capture image using a JEOL JEM-1400Plus transmission electron microscope or equivalent.
71. Analysis of images (i.e., size) can be done using ImageJ as described by Lam et al. (2021).^10^

#### Western Blot analysis

It is highly recommended that the isolated EVs are evaluated by Westen Blot analysis to confirm the presence of standard EV markers, such as tetraspanins (CD9, CD63, CD81), Alix, and/or Tsg101 (Figure 4C).

72. Elute large and small EVs from the columns (see step #41) in 65 μL Western Blot Lysis Buffer.
73. Place on ice for 45 min to allow complete solubilization of vesicles.
74. Save 5 μL from each sample to assess protein concentration using a Bradford Assay.
75. Dilute samples in 4× LDS sample buffer (Novex by Life Technologies) and DTT (50 mM final concentration) to the sample, ensuring thorough mixing by vortexing.
76. Heat at 95°C for 5 min in a heat block to denature proteins.
77. After heating, briefly spin tubes in a microcentrifuge.
78. Assemble polyacrylamide gel(s) in the electrophoresis apparatus and fill with NuPAGE MOPS SDS Running Buffer.
79. Load molecular weight marker and samples onto the gel with appropriate percentage for the molecular weights of the proteins of interest.

***Note:*** We use the Invitrogen NuPAGE Novex gel system and we find that pre-made 12% gels allows for separation of the standard EV proteins. We also usually load 20–30 μg/well of heart lysates and EVs.

80. Run gel at 180 V for about 60 min or until the dye reaches the bottom of the gel.
81. Transfer proteins onto a nitrocellulose membrane.
  a. Soak sponges, Whatman filter paper and the nitrocellulose membrane in transfer buffer.
  b. Prepare the transfer sandwich by layering the components. From bottom to top: 3 sponges → 2 filter papers → membrane → gel → 2 filter papers → 3 sponges.
  c. Place the sandwich into the transfer cassette.
  d. Fill the cassette with cold transfer buffer and partially fill the transfer box with additional transfer buffer.
82. Transfer for 150 min at 30 V at 4°C (in a cold room or refrigerator).
83. When the transfer is complete, stain the membrane with Ponceau S for 5 min, then rinse in ddH_2_O.
84. Capture a picture of the total proteins before completely rinsing off the stain with Tris-buffered saline+0.05% Tween 20 (TBST).

***Note:*** The Ponceau S stain allows for staining of total protein levels and can be used to compare the level of EV secretion between samples. This can be useful if comparing two conditions such as young vs aged tissues.

85. Block membrane with TBST+5% non-fat milk for 1 h at 18°C–20°C.
86. Incubate the membrane with the primary antibody against EV markers of choice for 12–18 h at 4°C on a rocker.

***Note:*** Primary antibodies are usually prepared at 1:1000 in TBST+5% non-fat milk. The optimal incubation time will depend on the antibody and should be determined empirically.

87. The next morning, wash the membrane 3 × 10 min with TBST on a rocker at 18°C–20°C.

***Note:*** The washing steps are important to reduce non-specific background. Also, it is important to make sure that the membrane remains moist at all steps.

88. Incubate membrane with the appropriate secondary antibody for 1 h at 18°C–20°C.

***Note:*** Secondary antibodies are usually prepared at 1:5000 in TBST+5% non-fat milk.

89. Wash the membrane 3 × 10 min with TBST on a rocker at 18°C–20°C.
90. Add a substrate for detecting the antibody bound to the protein.

***Note:*** We use Enhanced chemiluminescence (ECL) (Thermo Fisher Scientific) for detection of proteins.

91. Visualize and capture proteins on the membrane in a ChemiDoc Imaging System or equivalent.

## Expected outcomes

It is common practice to isolate and characterize EVs that are circulating in the plasma from animal models and patients. However, we and others have discovered that EVs secreted by cells do not always enter the circulation but are retained in the tissue.^1^^,^^11^ In addition, most studies to date have focused on small EVs (also known as exosomes), but it is now clear that cells also release larger EVs that can differ in cargo content.^1^ Here, we describe a protocol that allows for the separation of both large and small EVs from interstitial space of cardiac tissue through enzymatic digestion. The protocol can be used for either mouse hearts or for frozen cardiac tissues previously collected from patients. This protocol can also be adapted for use in other tissues and cell culture models. A successful separation procedure from 100 mg of heart tissue usually yields ∼1 × 10^12^–2 × 10^13^ of small EVs and ∼2 × 10^11^–2 × 10^12^ of large EVs for various experiments, including proteomics analysis, fluorescence microscopy, molecular and biochemical analysis. Characterization of EVs isolated from healthy versus diseased subjects can provide insights into their potential roles in various diseases such as cancer and neurodegeneration.^12^^,^^13^ The isolated EVs can also be used to study their therapeutic potential *in vitro* and *in vivo*.^14^^,^^15^ Moreover, the first time this protocol is utilized on a new model, the size distribution and yield should be evaluated by NTA (Figure 4A) and negative staining EM (Figure 4B). The size for large EVs ranges from ∼200–800 nm, whereas the size for small EVs is ∼50–200 nm. EVs are used within 1–2 h after their separation for most experiments, including NTA and negative stain EM. Exception are the Western Blot and proteomic analyses since proteins are stable at −20°C. Western Blot analysis of the isolated EVs is also recommended to confirm the presence of various EV markers, including CD63, CD81, Alix, and/or Tsg101 (Figure 4C).^16^ These markers were selected because they are present on the surface and inside of EVs.^16^^,^^17^ Cardiac EVs are also enriched in Troponin T which can be used as a marker in the Western Blot to confirm the separation of EVs secreted by cardiac myocytes.^18^ In addition, proteomic analysis is valuable in identifying the composition of the EVs and their cargo. This allows investigators to evaluate if the cargo is different in disease.

## Limitations

The advantage of our protocol is that it allows for the separation of large and small EVs by combining two different common methods to isolate EVs. Another benefit is that our protocol also describes how to release EVs from human heart tissue which can be utilized to study EVs in patients diagnosed with cardiovascular disease. The advantage of combining filtration, differential centrifugation and immunoaffinity purification is that it will yield a purer fraction of EVs compared to using differential centrifugation alone. However, the multiple steps in our protocol can also lead to lower yield which is the major limitation of this protocol. Thus, it is difficult to obtain enough EVs from one heart that can be used to perform multiple experiments. Moreover, our separation protocol uses antibodies against 3 different surface markers (CD81, CD63, and CD9) to maximize the yield of EVs. Recent studies have reported that subpopulations of EVs exists.^19^^,^^20^ Our protocol does not distinguish between different types of EVs and represents a mixture of different subpopulations. Also, the standard EV markers are present on EVs originating both from late endosomes/multivesicular bodies (MVB) and from budding of the plasma membrane.^16^ Specific markers that can be used to distinguish EVs of different cellular origin are still lacking. In addition, a clear separation between small and large EVs cannot be guaranteed since there exists an overlap between small and large EVs that are 150–200 nm in size. Thus, the possibility of contamination exists, with the potential for large EVs to be present in the small EV pellet and vice versa. Finally, there are no known specific cell surface markers for EVs secreted by cardiac myocytes that can be used for separation of EVs. However, genetic mouse models with tissue specific expression of fluorescently tagged CD63 when crossed with lineage-specific Cre recombinase driver mice have been developed and allows for tracing and separation of tissue specific EVs *in vivo*.^21^^,^^22^

## Troubleshooting

### Problem 1

Insufficient digestion of cardiac tissue (Step 14).

### Potential solution

Diseased hearts are often very fibrotic and we have noticed that increased digestion time is required to release the EVs from the tissue. We found that extending digestion time of human cardiac tissues from heart failure patients undergoing heart transplants to 60 min results in a higher yield of isolated EVs. However, it is important to maintain the same digestion time across comparable groups.

### Problem 2

Resuspending the small EV pellet after ultracentrifugation can be difficult (Step 31).

### Potential solution

We have observed that sometimes it is challenging to resuspend the small EV pellet from the final ultracentrifugation step. This problem is more common when isolating EVs from frozen tissue. We have observed that it helps if the pellet is initially resuspended using a reduced volume of PBS. A smaller volume of PBS in the tube generates more force to help break up the pellet. We initially resuspend the small EV pellet in 50 μL of PBS, followed by flicking of tube and repeated pipetting of the pellet. Once the pellet has completely dissolved in this smaller volume, the total volume can then be adjusted to 500 μL with additional PBS.

### Problem 3

Low yield of EVs (Step 41).

### Potential solution

The number of EVs secreted by cells is in general low and it can be challenging to isolate enough EVs for more than one experiment. It means that a separate EV preparation is required for each experiment and that it is not possible to validate findings with multiple experiments using EVs from the same heart. In general, each purification step will lower the yield of EVs. To minimize loss of EVs during both separation and immunoaffinity purification, we suggest the following: 1) work swiftly throughout the procedure; 2) always keep buffers chilled and samples on ice; 3) process a smaller number of hearts at a time to prevent delays between steps. Alternatively, it is possible to pool hearts from the same group and scale up the reagents used in this protocol. This approach can help ensure that you obtain enough EVs for more than one experiment.

### Problem 4

Presence of salt crystals and other artifacts in negative stain (Step 71).

### Potential solution

Uranyl acetate can form precipitates with phosphate. Consider carefully rinsing the EVs adhering to the EM grid with additional ddH_2_O before staining with uranyl acetate. Also, be careful not to allow the EVs to dry between washing and staining because this can create artifacts.

### Problem 5

Low transfer efficiency of proteins to the nitrocellulose membrane (Step 82).

### Potential solution

Make sure to use new transfer buffer for each transfer. Consider extending or reducing transfer time or voltage. Large proteins transfer more slowly while small proteins transfer more quickly. Use a roller to ensure that all air bubbles are eliminated in the transfer sandwich. Air bubbles in the sandwich during transfer will lead to blank spots on the nitrocellulose membrane. Also, it is important to make sure that the membrane stays wet in transfer buffer both before and after transfer.

## Resource availability

### Lead contact

Further information and requests for resources and reagents should be directed to and will be fulfilled by the lead contact, Asa B Gustafsson (abgustafsson@health.ucsd.edu).

### Technical contact

Technical questions on executing this protocol should be directed to and will be answered by the technical contact, Wenjing Liang (wel257@health.ucsd.ed)

### Materials availability

This study did not generate new unique reagents.

### Data and code availability

This study did not generate new Data sets or Code.
